# Multi-cellular natural killer (NK) cell clusters enhance NK cell activation through localizing IL-2 within the cluster

**DOI:** 10.1038/srep40623

**Published:** 2017-01-11

**Authors:** Miju Kim, Tae-Jin Kim, Hye Mi Kim, Junsang Doh, Kyung-Mi Lee

**Affiliations:** 1 School of Interdisciplinary Bioscience and Bioengineering (I-Bio), Pohang University of Science and Technology, Pohang, Gyeongbuk 790-784, Korea; 2 Amore-Pacific R&D Centre, Yongin, 17074, Korea; 3 Global Research Lab, Department of Biochemistry and Molecular Biology, Korea University College of Medicine, Seoul 136-713, Korea; 4 Division of Integrative Biosciences and Biotechnology (IBB), Pohang University of Science and Technology, Pohang, Gyeongbuk 790-784, Korea; 5 Department of Mechanical Engineering, Pohang University of Science and Technology, Pohang, Gyeongbuk 790-784, Korea; 6 Department of Melanoma Medical Oncology and Immunology, MD Anderson Cancer Center, Houston, Texas 77054, United States

## Abstract

Multi-cellular cluster formation of natural killer (NK) cells occurs during *in vivo* priming and potentiates their activation to IL-2. However, the precise mechanism underlying this synergy within NK cell clusters remains unclear. We employed lymphocyte-laden microwell technologies to modulate contact-mediated multi-cellular interactions among activating NK cells and to quantitatively assess the molecular events occurring in multi-cellular clusters of NK cells. NK cells in social microwells, which allow cell-to-cell contact, exhibited significantly higher levels of IL-2 receptor (IL-2R) signaling compared with those in lonesome microwells, which prevent intercellular contact. Further, CD25, an IL-2R α chain, and lytic granules of NK cells in social microwells were polarized toward MTOC. Live cell imaging of lytic granules revealed their dynamic and prolonged polarization toward neighboring NK cells without degranulation. These results suggest that IL-2 bound on CD25 of one NK cells triggered IL-2 signaling of neighboring NK cells. These results were further corroborated by findings that CD25-KO NK cells exhibited lower proliferation than WT NK cells, and when mixed with WT NK cells, underwent significantly higher level of proliferation. These data highlights the existence of IL-2 trans-presentation between NK cells in the local microenvironment where the availability of IL-2 is limited.

Natural killer (NK) cells are innate immune cells that participate in tumor surveillance and pathogen clearance by killing transformed/infected cells and producing multiple cytokines^1^,^2^. NK cells are activated when they recognize down-regulation of the class I major histocompatibility complex (MHC-I) or overexpression of ligands for their activation receptors such as NK1.1, NKG2D, NKp46, 2B4, DNAM-1, and natural cytotoxicity receptors (NCRs)^3^,^4^. Cytokines such as IL-2, IL-12, IL-15, IL-18, and type I interferons (IFNs) also contribute to NK cell priming and expansion^5^. Although the molecular signals involved in NK cell activation are known, the detailed cellular contexts providing such signals are not completely understood because of the complexities of *in vivo* microenvironments where NK cell activation occurs.

NK cell priming mostly occurs in secondary lymphoid organs where many cells are densely packed^6^,^7^. Dendritic cells (DCs) play a major role in NK cell priming by secreting stimulatory cytokines and presenting ligands for activating receptors^8^,^9^. In addition to providing stimulatory signals to NK cells, activated DCs produce chemokines to recruit NK cells and other immune cells such as granulocytes, monocytes, and T cells, which cause nucleation of multi-cellular clustering^10^,^11^. Complex intercellular interactions in such multi-cellular clusters may synergize and coordinate immune responses, but at the same time, immune cells may also compete with each other for the limited supply of cytokines. For example, CD4 + T cells, CD8 + T cells, regulatory T cells (Tregs), and NK cells all require IL-2 for their activation and proliferation, but Tregs, which constitutively express high-affinity IL-2 receptors (IL-2Rs), consume large amounts of IL-2 to limit the accessible amounts of IL-2^12^,^13^,^14^,^15^. Formation of multi-cellular clusters can not only promote interactions among different cell types, but also increase the probability of interactions among identical cells, or homotypic cell-to-cell interactions^16^,^17^. Indeed, homotypic interactions among activating lymphocytes such as CD4 + T cells, CD8 + T cells, and NK cells during priming have been shown to promote activation and differentiation of lymphocytes^18^,^19^,^20^.

In this study, we dissected the mechanism of contact-mediated homotypic interactions among NK cells that augmented IL-2 signaling. We employed lymphocyte-laden microwell technologies, which allow precise control of contact-mediated interactions among lymphocytes and quantitative fluorescence imaging of single cells^21^,^22^. Characterization of phosphorylation, expression and polarization of signaling molecules within multi-cellular clusters of NK cells revealed that IL-2 captured by IL-2R on one NK cell could trigger IL-2R signaling of other surrounding NK cells through intercellular contact. This IL-2 trans-presentation within multi-cellular clusters of NK cells can serve as an important strategy for NK cells to maximally utilize IL-2, which can be a limited resource during the early stages of immune responses because of the competition among many other types of lymphocytes.

## Results

### Experimental settings to quantitatively assess IL-2 mediated activation of NK cells

**Figure 1:**
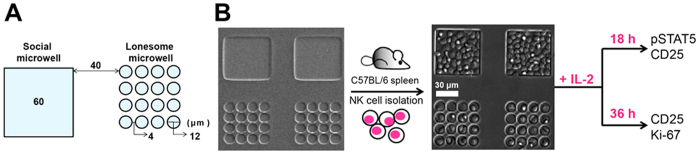
Schematic illustration of experimental settings. (**A)** Dimensions of social and lonesome microwells. (**B)** Experimental scheme for NK cell-laden microwell-based IL-2 stimulation assays.

To quantitatively assess multi-cellular interaction dependent IL-2 signaling in NK cells, culture dishes containing two different types of NK cell-laden microwells were fabricated (Fig. 1A)^21^,^22^. NK cells in a social microwell can exhibit contact-mediated interactions, whereas those in lonesome microwells cannot. Further, both social and lonesome microwells are located adjacent within the same dish so that NK cells in social or lonesome microwells are exposed to identical bulk media. Experiments using NK cell-laden microwells were performed as shown in Fig. 1B. First, NK cells purified from the spleens of C57BL/6 mice were seeded into microwells (left panel of Fig. 1B). The NK cells in the microwells were then activated with IL-2 for 18 or 36 h, fixed and stained with fluorophore labeled antibodies, and imaged using a fluorescence microscope. Typically, 25 planes of z-section images with 0.5 μm intervals were acquired and integrated into a single plane for visualization and further quantification.

### Enhanced IL-2 signaling of NK cells *via* contact-mediated multi-cellular interactions

**Figure 2:**
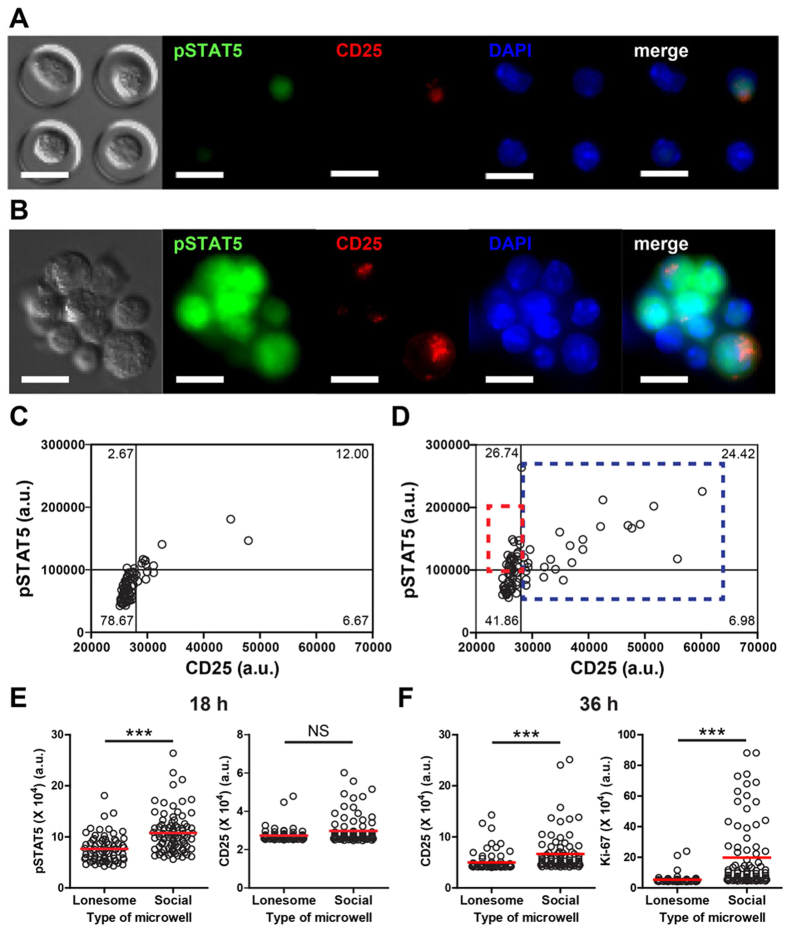
Single cell-based quantitative analysis of NK cells in microwells revealed that contact-mediated interactions significantly enhanced IL-2 signaling in NK cells. (**A,B**) Representative fluorescence images of NK cells in lonesome (**A**) and social (**B**) microwells. NK cells in microwells were stimulated with IL-2 for 18 h, fixed, stained, and imaged. Scale bar: 10 μm. (**C,D**) CD25 vs. pSTAT5 fluorescence intensity (arbitrary unit, a.u.) of NK cells in lonesome (**C**) and social (**D**) microwells. Individual dots represent the fluorescence intensity of individual cells. Red box: CD25^low^pSTAT5^high^ population; blue box: CD25^high^ population. N = 86 (social) and 75 (lonesome). (**E**) pSTAT5 and CD25 fluorescence intensity comparison between NK cells in lonesome vs. social microwells. (NK cells were stimulated with IL-2 for 18 h.) N = 86 (social), and N = 86 (lonesome). (**F**) CD25 and Ki-67 fluorescence intensity comparison between NK cells in lonesome vs. social microwells. (NK cells were stimulated with IL-2 for 36 h.) N = 83 (social), and N = 83 (lonesome). Data are representative of three independent experiments. Error bars: standard deviation. The Mann-Whitney test was performed. NS: not significant, * p < 0.05, **p < 0.01, ***p < 0.001.

Resting NK cells constitutively express dimeric intermediate-affinity IL-2R comprising CD122, a β chain of IL-2R, and CD132, a common γc receptor, which mediate IL-2 signaling. Upregulation of CD25 in NK cells results in the formation of trimeric high-affinity receptor (CD25/122/132), which further enhances IL-2 signaling^23^,^24^,^25^. Upon binding IL-2, IL-2R triggers signal transduction pathways that phosphorylate Signal Transducer and Activator of Transcription 5 (pSTAT5), and express CD25, an α chain of IL-2R. Therefore, we assessed if the multi-cellular cluster formation of NK cells affected STAT5 phosphorylation and CD25 expression in response to IL-2. Representative images of NK cells in social and lonesome microwells and quantification of fluorescence intensity of individual cells in each microwell are shown in Fig. 2A–D. In case of the NK cells in lonesome microwells, express levels of CD25 correlated well with the phosphorylation of STAT5 (Fig. 2A and C). The NK cells within social microwells frequently formed small multi-cellular clusters (Fig. 2B). Moreover, a fraction of cells in the clusters expressed high levels of CD25, whereas the majority of cells within the clusters exhibited high levels of pSTAT5, indicating that pSTAT5 levels within multi-cellular aggregates are enhanced by contact-mediated interactions among activating NK cells. Quantitatively, the majority of CD25^high^ NK cells in social microwells (a blue dashed rectangle region in Fig. 2D) were pSTAT5^high^, and expression levels of CD25 in CD25^high^ NK cells positively correlated with the levels of pSTAT5, similar to those in lonesome microwells (Fig. 2C). However, a significant fraction of CD25^low^ NK cells in social microwells expressed high levels of pSTAT5 (a red dashed rectangle region in Fig. 2D), suggesting contact-mediated cooperativity exist in social microwells. Overall, the fluorescence intensity of pSTAT5 in NK cells in social microwells was significantly higher than that of NK cells in lonesome microwells, although the fluorescence intensity of CD25 staining in NK cells in social microwells was not significantly different compared with that of NK cells in lonesome microwells 18 h after IL-2 stimulation (Fig. 2E). These results further support conclusion that enhanced STAT5 phosphorylation in NK cells in social microwells is induced by the contact-mediated incorporation among NK cells rather than the formation of trimeric IL-2R through CD25 upregulation. At 36 h after IL-2 addition, significantly higher levels of CD25 and Ki-67 were detected in NK cells in social microwells compared with those in lonesome microwells (Fig. 2F). These data indicate that enhanced NK cell expression of pSTAT5 in social microwells at 18 h induced marked augmented expression of CD25 and Ki-67 by NK cells in social microwells at 36 h. Moreover, IL-2-stimulated murine NK cells produced large amounts of IFN-γ, but almost undetectable amounts of other cytokines such as IL-6, IL-10, IL-12, IL-15, and IL-21 (Fig. S1 in Supplementary Information (SI)). Therefore, STAT5 phosphorylation and proliferation shown in Fig. 2 are likely mediated by exogenous IL-2 rather than by endogenous cytokines secreted by IL-2-stimulated NK cells.

### NK cells in social microwells polarized CD25, lytic granules, and microtubule organizing center (MTOC) toward neighboring NK cells

**Figure 3:**
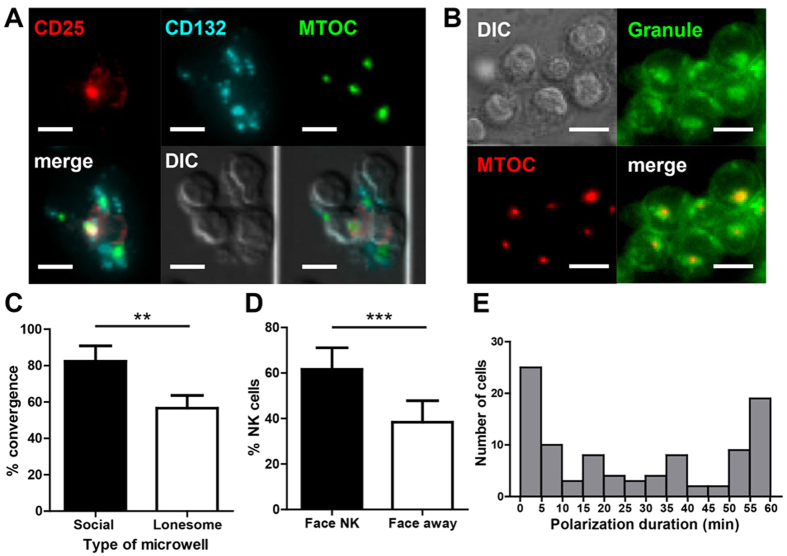
NK cells in social microwells polarized CD25, MTOC, and lytic granules toward neighboring NK cells. (**A)** Representative images showing the distributions of CD25, CD132, and the MTOCs of NK cells in social microwells. (**B)** Representative images showing the distributions of lytic granules and the MTOCs of NK cells in social microwells. (**C)** Quantification of lytic granule convergence to the MTOC. N = 248 (social), and N = 60 (lonesome) (**D)** Percentage of NK cells exhibiting lytic granules pointing toward neighboring NK cells (Face NK; black bar) or pointing away from neighboring NK cells (Face away; white bar). N = 258. (**E)** Distribution of lytic granule polarization durations. N = 200. (**A,B**) Scale bar: 10 μm. (**C,D**) Data are representative of three independent experiments. Error bars: standard deviation. The Mann-Whitney test was performed. **p < 0.01, ***p < 0.001.

To better understand how contact-mediated interactions among NK cells enhanced IL-2 signaling, the distribution of IL-2 receptors in NK cells within social microwells was examined along with microtubule organizing center (MTOC), which was visualized by pericentrin or tubulin immunostaining (Fig. 3A). CD132 formed multiple clusters of relatively small sizes, as previously reported^22^. In sharp contrast, the majority of CD25 was polarized toward one side of the NK cells as a single patch, and ~85% of polarized CD25 (64 out of 75) overlapped with MTOC, indicating polarization of CD25 toward the direction of the MTOC.

Typically, lymphocytes such as T cells and NK cells polarize their MTOC toward their interaction partners^16^,^26^,^27^,^28^. In particular, NK cells polarize the MTOC toward tumor cells along with lytic granules that induce tumor cell lysis^26^,^28^. Lytic granules are lysosome-related organelles in NK cells containing molecules required for cytolytic activity, including perforin and granzyme B. Lytic granules uniformly distributed in resting NK cells converge to MTOC by IL-2 stimulation^29^. To test whether granule convergence also occurs in our experimental setting, LysoSensor™ Green DND-189 was used to label lytic granules in NK cells^30^,^31^. The majority of NK cells in social microwells exhibited convergence of lytic granules toward the MTOC (Fig. 3B). In contrast, a significantly lower fraction of NK cells in lonesome microwells exhibited granule convergence compared with those in social microwells (Fig. 3C). These findings indicate that IL-2 induced convergence of lytic granules can be augmented by homotypic contact-mediated NK-NK interactions, presumably through interactions of activating receptor-ligand pairs co-expressed on the NK cell surfaces. LFA-1, which accumulates at NK-NK contact sites^22^, was partially responsible for the granule convergence because anti-LFA-1 blocking significantly reduced granule convergence of NK cells in social microwells (Fig. S2 in SI), similar to NK-target interactions^29^. Moreover, the analysis of the granule polarization of NK cells on the periphery of NK cell clusters revealed that the lytic granules that converged to the MTOC in NK cells mostly polarize toward neighboring NK cells (Fig. 3D), similar to multi-cellular clusters of activating T cells^18^, suggesting that active crosstalk among NK cells occurred in social microwells.

Next, we investigated the dynamics of NK cell polarization in social microwells by performing time-lapse imaging. NK cells in microwells were labeled with LysoSensor™ Green DND-189 for 1 h prior to time-lapse imaging. LysoSensor™ Green DND-189 can label lytic granules in live NK cells, thus can be used for live cell imaging. While approximately 75% NK cells exhibited prolonged (>5 min) granule polarization toward neighboring NK cells (Fig. S3A in SI), approximately 25% NK cells exhibited transient (<5 min) convergence/polarization of lytic granules (Fig. S3B in SI). As shown in Fig. 3E, the duration of granule polarization was evenly and broadly distributed between 5~55 min in approximately one-half of the NK cells, while approximately 20% NK cells polarized toward only one cell for >60 min. Moreover, approximately 60% NK cells exhibited >30 min of granule polarization toward neighboring NK cells, indicating dynamic and prolonged contact-mediated intercellular communication between neighboring NK cells. Importantly, we did not observe any degranulation and NK cell killing during time-lapse imaging, indicating that homotypic NK-NK interactions is sufficient for granule convergence and polarization, but not for degranulation^32^. Taken together, NK cells in social microwells polarized CD25, lytic granules, and MTOC toward neighboring NK cells during IL-2 stimulation.

### Enhanced proliferation of CD25 KO NK cells through contact-mediated help by WT NK cells

**Figure 4:**
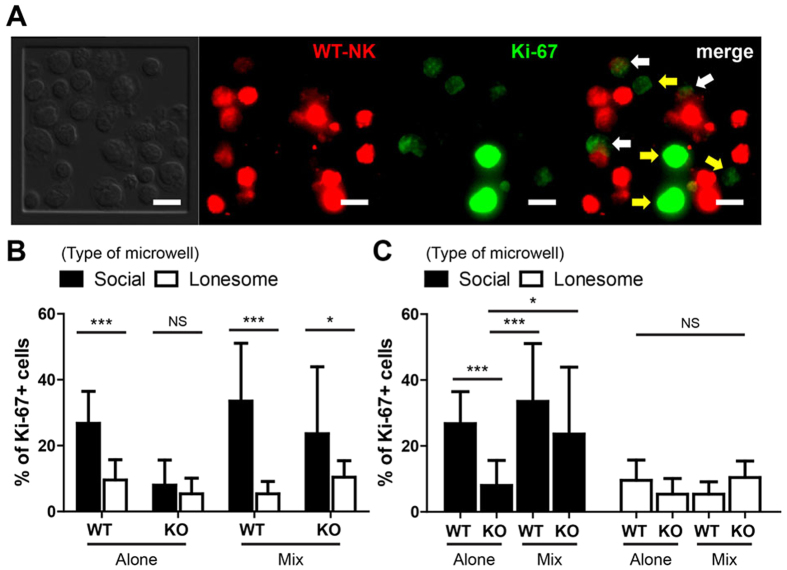
Proliferation of CD25KO NK cells was significantly enhanced when they were in contact with WT NK cells indicating that IL-2 trans-presentation occurred within multi-cellular clusters of NK cells. Microwell experiments using WT NK cells alone (WT-alone), CD25KO NK cells alone (CD25KO-alone), and a 50:50 mixture of WT (WT-mix) and CD25KO NK cells (CD25KO-mix) were performed, and the percentage of Ki-67 + NK cells were measured using immunofluorescence microscopy. For each case, N > 300 for social microwells, and N > 150 for lonesome microwells. (**A**) Representative images showing fluorescence staining of WT NK cells and Ki-67 in MIX experiments. White arrows: Ki-67 + WT NK cells. Yellow arrows: Ki-67 + CD25KO NK cells. Scale bar: 10 μm. (**B,C**) Percentage of Ki-67 + NK cells in each experimental condition. Statistical analysis is conducted for the same type of cells in different types of microwells (**B**) or different types of cells in the same type of microwell (**C**). Data are representative of three independent experiments. Error bars: standard deviation. The Mann-Whitney test was performed for pair-wise comparison, and one-way ANOVA test was performed for group comparison. NS: not significant, *p < 0.05, ***p < 0.001.

Dynamic polarization of CD25 toward neighboring NK cells in the presence of IL-2 may result in trans-presentation of IL-2; IL-2 bound on one NK cell expressing high levels of CD25 can trigger IL-2 signaling of the neighboring NK cells that only express CD122/CD132. In this way, IL-2-mediated activation of NK cells in social microwells can be significantly enhanced. To test this possibility, we performed microwell experiments using NK cells isolated from the spleens of wild type (WT) or CD25 knock-out (KO) mice. Three different experimental conditions were considered: (1) WT NK cells alone (WT-alone), (2) CD25 KO NK cells alone (CD25 KO-alone), and (3) a 50:50 mixture of WT and CD25 KO NK cells (mix). NK cells in microwells stimulated with IL-2 for 36 h were fixed, and Ki-67 expression was measured using immunofluorescence microscopy. For ‘mix’ case, WT cells were labeled with DDAO-SE, a far red fluorescent dye that distinguishes Ki-67 expression in WT-mix from that of CD25 KO-mix NK cells (Fig. 4A). The percentage of Ki-67+ cells was measured by counting NK cells emitting Ki-67 fluorescence higher than the background threshold values (Fig. 4B and C). As expected, a significantly higher percentage of WT-alone NK cells in social microwells expressed Ki-67 compared with WT-alone NK cells in lonesome microwells (Fig. 4B). In contrast, Ki-67 expression of CD25 KO-alone NK cells in social microwells was comparable with that of CD25 KO-alone NK cells in lonesome microwells (Fig. 4B), indicating that CD25 plays an important role in synergistic IL-2 signaling among NK cells in social microwells. Moreover, Ki-67 expression of CD25 KO-mix NK cells in social microwells was comparable to that of WT-mix NK cells in social microwells, and significantly higher than that of CD25 KO-alone NK cells in social microwells (Fig. 4C). These results highlight that CD25 expressed on WT NK cells augment the proliferation of neighboring CD25 KO NK cells by securing IL-2 on CD25 and allow trans-presentation of IL-2.

## Discussion

In this study, we uncover the existence of IL-2 trans-presentation within multi-cellular NK clusters that synergizes with IL-2R signaling at the membrane proximal step. Small fraction of NK cells expressing CD25 can capture IL-2 on their surface CD25 and present it to heterodimeric CD122/CD132, intermediate-affinity IL-2R, of neighboring NK cells lacking CD25 to facilitate their activation. Considering that NK cell priming *in vivo* occurs in the presence of abundant natural Tregs, which consume considerable IL-2 by constitutively expressing CD25, such a CD25/IL-2 trans-presentation mechanism secures the IL-2 from shared limited resources. Indeed, Tregs and NK cells *in vivo* compete each other to acquire IL-2 as previously shown; depletion of Tregs in mice significantly enhances cytotoxicity of NK cells^12^, and suppressing the IL-2 reception of Tregs by anti-CD25 mAbs leads to the proliferation of CD56^bright^ NK cells in humans^13^.

Activation of NK cells by cytokine trans-presentation has been mainly studied using IL-15;^8^,^33^,^34^,^35^ IL-15 bound on an IL-15 receptor α chains (IL-15Rα of various cells such as activated monocytes, dendritic cells, and cancer cells have shown to enhance proliferation and cytotoxicity of NK cells. Moreover, IL-2R and IL-15R comprise identical β and γc chains(CD122 and CD132), and resting NK cells that constitutively express β and γc chains of IL-2/15 R can readily receive IL-2/15 trans-presented by other cells^16^,^25^. In case of IL-15, IL-15Rα binds with exceptionally high affinity to IL-15 (Kd ~ 10 pM^36^). Thus, IL-15 can remain bound on IL-15Rα for a prolonged period of time. In contrast, the binding affinity of IL-2/CD25 is approximately 1000-fold lower than that of IL-15/IL-15Rα (Kd ~ 10 nM^24^,^37^); thus, IL-2 bound on CD25 will be quickly dissociated. Therefore, IL-2 trans-presentation can only occur shortly after IL-2/CD25 binding, therefore, may require pre-engagement of cells, or at least their close proximity^16^,^38^.

Indeed, DCs trans-present IL-2 to T cells in the context of the immunological synapse where tight cell-to-cell contact forms and directed secretion of IL-2 occurs^39^. Polarized secretion of IL-2 and enhanced phosphorylation of STAT-5 in the synapse formed between activating T cells, which indicates trans-presentation of IL-2 among activating T cells^18^. Importantly, in both situations, IL-2 is produced by at least one cell, either DCs or T cells, that participates in the immunological synapse. In contrast, IL-2 trans-presentation among activating NK cells solely relies on IL-2 secreted by other cells such as DCs or T cells, because NK cells do not produce IL-2. Therefore, to efficiently trans-present IL-2 among activating NK cells, multi-cellular cluster formation of NK cells as well as close positioning of NK cell clusters near DCs and T cells is required. Indeed, multi-cellular clustering of NK cells is typically nucleated by DCs in T cell-rich areas of secondary lymphoid organs^10^,^11^, indicating that IL-2 secreted by DCs or T cells is maximally utilized through trans-presentation from nearby NK cells.

Preferential polarization of CD25, MTOC, and lytic granules toward NK-NK interfaces demonstrated in the present study suggests that homotypic NK-NK interactions occur in the extended context of immunological synapses^16^. In addition to the polarization of CD25, MTOC, and lytic granules, activating receptors such as 2B4^20^,^22^ and LFA-1^22^ accumulate toward NK-NK interfaces. Detailed characterization and identification of the functional roles of homotypic NK-NK interactions under various physiological/pathological contexts would be an exciting future research topic.

During the early stage of IL-2 activation, NK-NK homotypic interactions significantly enhance IL-2 reception by 2B4-mediated CD122/132 clustering^22^, and IL-2 trans-presentation by CD25. Because knocking out of either 2B4 or CD25 is sufficient to completely abrogate contact-mediated enhancement of IL-2 signaling, CD122/132 clustering and IL-2 trans-presentation by CD25 serves an essential role in facilitating NK cell responses to IL-2. IL-2 trans-presentation occurring within multi-cellular clusters of NK cells ensures the availability of IL-2, which can be a limited resource during the early stages of immune responses.

## Materials and Methods

### Mice and NK cell isolation

C57BL/6 mice were purchased from the POSTECH Biotech Center (PBC) animal facility, and CD25^−/−^ mice were purchased from the Jackson Laboratory. The mice were bred and housed under specific pathogen-free conditions in the PBC animal facility. All methods involving mice were carried out in accordance with the guidelines of the Institutional Animal Care and Use Committee at the PBC, and all experimental protocols were approved by the Institutional Animal Care and Use Committee at PBC. NK cells were isolated from the spleens of C57BL/6 wild type or CD25^−/−^ mice using a magnetic NK cell isolation kit (Miltenyi Biotec).

### NK cell-laden microwell fabrication

Surfaces containing various sizes of microwells were fabricated as described previously^21^,^22^. Anti-CD44 (eBioscience, clone: IM7) was immobilized at the floors of the microwells to capture NK cells within microwells. Primary NK cells (3.75 × 10^7^ cells/mL) isolated from mice were added to the anti-CD44 functionalized microwells and incubated at 4 °C for 1 h with gentle shaking. Then, by gently washing the cell-seeded microwells with cold PBS, unattached cells were removed to form NK cell-laden microwells.

### Activation of NK cells in microwells

NK cells in microwells were cultured at 37 °C in a 5% CO_2_ humidified incubator in RPMI culture medium containing 10% FBS (GIBCO), 1% Penicillin/Streptomycin (Invitrogen) in the presence of recombinant murine IL-2 (50 ng/ml, Peprotech). For co-culture of WT and CD25 KO NK cells, WT NK cells were labeled with 10 μM of CellTrace™ Far Red DDAO-SE (Invitrogen).

### Immunofluorescence microscopy of NK cells in microwells

For staining of cell surface markers, cells in the microwells were washed twice with cold PBS and immediately fixed with 1% paraformaldehyde for 15 min at 4 °C. The fixed samples were stained with various antibodies in staining buffer (PBS containing 2% FBS and 0.01% sodium azide) for 1 h at room temperature. For intracellular staining, staining buffer containing 0.2% saponin (Sigma) was used. For staining of pSTAT5, cells were fixed using methanol. The following antibodies were used for staining: anti-pSTAT5 (Cell Signaling Technology, Danvers, MA, USA, Clone: Tyr694;C71E5), anti-Ki-67 (eBioscience, clone: B56), anti-CD25 (eBioscience, clone: PC61.5), anti-CD132 (eBioscience, clone: TUGh4), anti-tubulin (Molecular Probes), and anti-pericentrin (Abcam, Cambridge, MA, polyclonal). A modified Zeiss Axio Observer.Z1 epi-fluorescence microscope with a 40× (Plan-Neofluar, NA = 1.30) objective lens and a Roper Scientific CoolSnap HQ CCD camera was used for imaging. An XBO 75 W/2 Xenon lamp (75 W, Osram) and DAPI (EX. 365, BS 395, EMBP445/50), eGFP (EX BP 470/40, BS 495, EMBP 525/50), Cy3 (EX BP 550/25, BS 570, EMBP 605/70), and Cy5 (EX BP 620/60, BS 660, EMBP 770/75) filter sets were used for fluorescence imaging. Fluorescence images of fixed and stained NK cells were acquired using optical z-sectioning (25 individual planes, 0.5 μm apart). The integrated intensity of each molecule along the z-axis was obtained using ImageJ (NIH) and Metamorph (Molecular Devices).

### Cytokine detection

NK cells in 24 well tissue culture plate were stimulated with IL-2 (50 ng/ml) for 48 h. The concentration of IL-21 and IL-15 were measured in ELISA (eBiosciences) and IL-12, IL-6, IL-10, and IFN-γ were determined with mouse inflammation CBA Kit (BD Bioscience) according to manufacturer’s recommendations.

### Live cell imaging of lytic granule dynamics

To visualize lytic granules, NK cells in microwells were incubated with 1 μM LysoSensor™ Green DND-189 (Invitrogen, diluted in cell culture medium) for 30 min at 37 °C. For live cell imaging, a coverslip containing NK cell-laden microwells mounted into a Chamlide chamber (Live Cell Instrument, Korea) was placed on a microscope stage equipped with a Chamlide TC incubator system maintaining 37 °C and 5% CO_2_ (Live Cell Instrument, Korea). Time-lapse microscopy was conducted at 5-min intervals for 1.5 h, and DIC and green (lysosensor) fluorescence images were acquired in rapid succession at each time interval. For lysosensor imaging, three optical section images over 2.5 μm distances in the z-direction were acquired and integrated.

### Statistical analysis

All statistical analyses were performed using GraphPad Prism software. Results were presented as the means ± standard deviation (SD), and P < 0.05 (*), P < 0.01 (**), P < 0.001 (***) were considered significant.

## Additional Information

**How to cite this article**: Kim, M. *et al*. Multi-cellular natural killer (NK) cell clusters enhance NK cell activation through localizing IL-2 within the cluster. *Sci. Rep.* **7**, 40623; doi: 10.1038/srep40623 (2017).

**Publisher's note:** Springer Nature remains neutral with regard to jurisdictional claims in published maps and institutional affiliations.

## Supplementary Material

Supplementary Information
