# Supplementary material for: Multi-cellular natural killer (NK) cell clusters enhance NK cell activation through localizing IL-2 within the cluster
Source: Sci Rep. 2017 Jan 11;7:40623. doi: 10.1038/srep40623 (PMC5225448; doi:10.1038/srep40623)
Supplement: Supplementary Information [file srep40623-s1.pdf]

Supplementary Information

Multi-cellular natural killer (NK) cell clusters enhance NK cell activation through localizing IL-2 within the cluster

Miju Kim[1, 5], Tae-Jin Kim[2], Hye Mi Kim[3], Junsang Doh[1, 4, *] and Kyung-Mi Lee[2, *]

[1]School of Interdisciplinary Bioscience and Bioengineering (I-Bio), Pohang University of S cience and Technology, Pohang, Gyeongbuk 790-784, Korea

[2]Global Research Lab, Department of Biochemistry and Molecular Biology, Korea University College of Medicine, Seoul 136-713, Korea

[3]Division of Integrative Biosciences and Biotechnology (IBB), Pohang University of Scienc e and Technology, Pohang, Gyeongbuk 790-784, Korea

[4]Department of Mechanical Engineering, Pohang University of Science and Technology, Pohang, Gyeongbuk 790-784, Korea

[5]Amore-Pacific R&D Centre, Yongin, 17074, Korea

Address for correspondence:
*Kyung-Mi Lee, Ph.D: Department of Biochemistry and Molecular Biology, Korea University College of Medicine, Sungbook-Gu Anam-Dong 5Ga, Seoul, 136-705, Korea
e-mail: kyunglee@korea.ac.kr, phone: +82-2-920-6251, Fax +82-920-6252.

*Junsang Doh, Ph.D: School of Interdisciplinary Bioscience and Bioengineering (I-Bio), Pohang University of Science and Technology, Pohang, Gyeongbuk 790-784, Korea

e-mail: jsdoh@postech.ac.kr, phone: +82-54-279-2189, Fax: +82-54-279-3199.

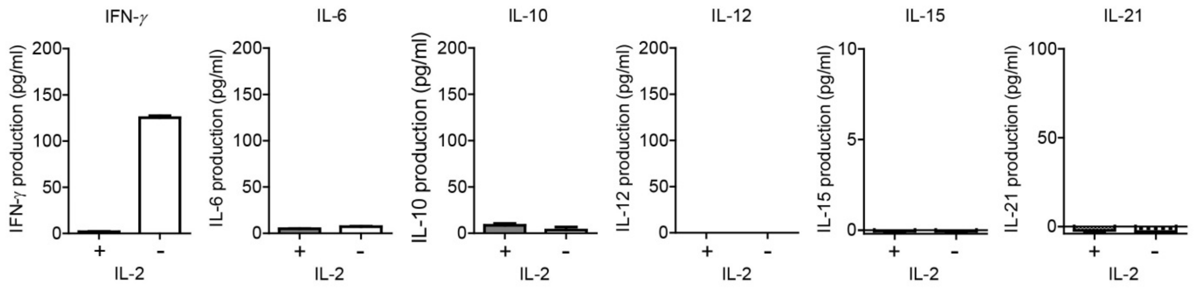

Figure S1. Cytokine secretion of IL-2-stimulated NK cells. NK cells were cultures in the presence (50 ng/ml) or absence of IL-2 for 48 h, and concentrations of various cytokines in the supernatant were measured.

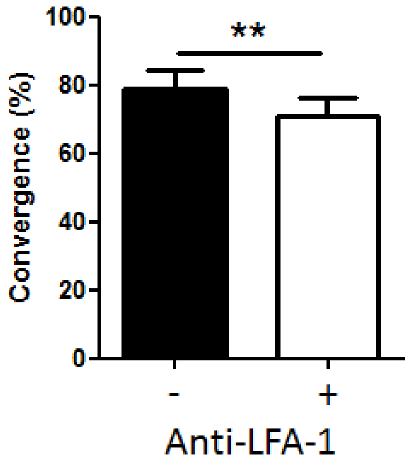

Figure S2. Effect of anti-LFA-1 treatment on granule convergence of NK cells in social microwells. NK cells in social microwells were stimulated with IL-2 in the presence (10 μg/ml) or absence of anti-LFA-1 (clone: M17/4) for 18 h, and granule convergence was assessed using LysoSensor™ Green DND-189. N = 380 (- anti-LFA-1), and N = 369 (+ anti-LFA-1). Error bars: standard deviation. The Mann-Whitney test was performed. ** p<0.01.

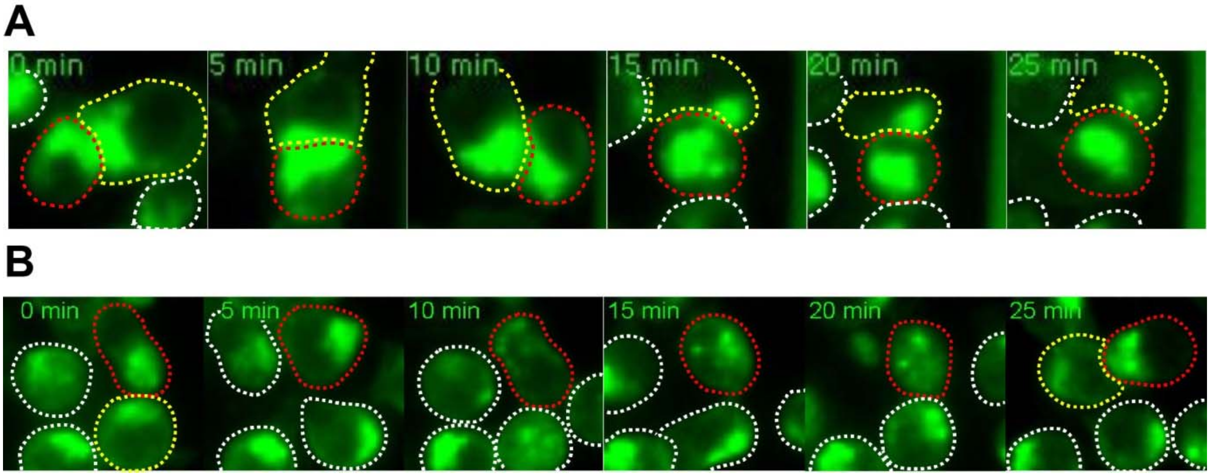

Figure S3. NK cells in social microwells exhibited dynamic polarization of lytic granules toward neighboring NK cells. A. Representative time-lapse images showing stable prolonged lytic granule polarization of a NK cell (a red dashed circle) toward a neighboring NK cells (a yellow dashed circle). Bystander NK cell boundaries are marked with white dashed circles. B. Representative time-lapse images showing transient lytic granule convergence/polarization.
